# A feasibility study of in vivo quantitative ultra-short echo time-MRI for detecting early cartilage degeneration

**DOI:** 10.1186/s13244-024-01734-4

**Published:** 2024-06-26

**Authors:** Xiaolian Su, Yitong Wang, Jieying Chen, Zonghui Liang, Lidi Wan, Guangyu Tang

**Affiliations:** 1 Department of Radiology, Shanghai Tenth People’s Hospital, School of Medicine, Tongji University, Shanghai, 200092 China; 2 Department of Radiology, Shanghai Stomatological Hospital & School of Stomatology, Fudan University, Shanghai, China; 3 Chongming Branch of Tenth People’s Hospital Affiliated to Tongji University, Shanghai, China; 4 Department of Radiology, Shanghai Jing’an District Central Hospital, Shanghai, China

**Keywords:** Cartilage degeneration, Ultrashort echo time (UTE), Magnetic resonance imaging, In vivo

## Abstract

**Objectives:**

To explore the feasibility of Ultra-short echo time (UTE) – MRI quantitative imaging in detecting early cartilage degeneration in vivo and underlying pathological and biochemical basis.

**Methods:**

Twenty volunteers with osteoarthritis (OA) planning for total knee arthroplasty (TKA) were prospectively recruited. UTE-MRI sequences and conventional sequences were performed preoperatively. Regions of interests (ROIs) were manually drawn on the tibial plateau and lateral femoral condyle images to calculate MRI values. Cartilage samples were collected during TKA according to the preset positions corresponding to MR images. Pathological and biochemical components of the corresponding ROI, including histological grading, glycosaminoglycan (GAG) content, collagen integrity, and water content were obtained.

**Results:**

91 ROIs from volunteers of 7 males (age range: 68 to 78 years; 74 ± 3 years) and 13 females (age range: 57 to 79 years; 67 ± 6 years) were evaluated. UTE-MTR (*r* = −0.619, *p* < 0.001), UTE-AdiabT1ρ (*r* = 0.568, *p* < 0.001), and UTE-T2* values (*r* = −0.495, *p* < 0.001) showed higher correlation with Mankin scores than T2 (*r* = 0.287, *p* = 0.006) and T1ρ (*r* = 0.435, *p* < 0.001) values. Of them, UTE-MTR had the highest diagnostic performance (AUC = 0.824, *p* < 0.001). UTE-MTR, UTE-AdiabT1ρ and UTE-T2* value was mainly related to collagen structural integrity, PG content and water content, respectively (*r* = 0.536, −0.652, −0.518, *p* < 0.001, respectively).

**Conclusion:**

UTE-MRI have shown greater in vivo diagnostic value for early cartilage degeneration compared to conventional T2 and T1ρ values. Of them, UTE-MTR has the highest diagnostic efficiency. UTE-MTR, UTE-AdiabT1ρ, and UTE-T2* value mainly reflect different aspects of cartilage degeneration--integrity of collagen structure, PG content, and water content, respectively.

**Critical relevance statement:**

Ultra-short echo time (UTE)-MRI has the potential to be a novel image biomarkers for detecting early cartilage degeneration in vivo and was correlated with biochemical changes of early cartilage degeneration.

**Key Points:**

- Conventional MR may miss some early cartilage changes due to relatively long echo times.
- Ultra-short echo time (UTE)-MRI showed the ability in identifying early cartilage degeneration in vivo.
- UTE-MT, UTE-AdiabT1ρ, and UTE-T2* mapping mainly reflect different aspects of cartilage degeneration.

**Graphical Abstract:**

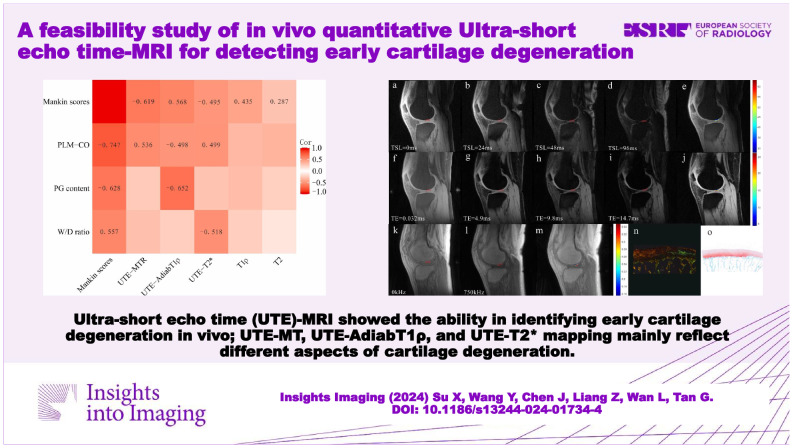

## Introduction

Osteoarthritis (OA) is a global joint disease characterized with cartilage degeneration which leads to pain, functional impairment, and reduced quality of life for affected individuals [1]. The primary components of cartilage consist of water, collagen, and proteoglycan (PG), which play a crucial role in the progression of cartilage degeneration [1, 2].

In clinical studies, non-invasive conventional MRI protocols such as T1ρ, T2 mapping, and T2* mapping are often used to detect early cartilage degeneration [3]. However, they may not capture signals from all layers of cartilage, especially the calcified and deep layers, due to their relatively long echo times. UTE sequences have been developed and increasingly employed to overcome the aforementioned shortcomings, with echo times as short as 8 μs [4]. Previous in vivo and in vitro studies have investigated the applicability of UTE sequences for assessing cartilage, as well as the relationship between UTE-MRI parameters and alterations in biochemical components such as collagen structure integrity, PG content, and water content during early cartilage degeneration [5–10]. However, some limitations existed such as (a) the use of cartilage from non-human sources [11, 12]; (b) conducting the research in vitro [5, 7]; (c) relying solely on imaging evaluation without sufficient pathological component analysis [6, 10]; or (d) only utilizing one or two UTE-MRI sequences to evaluate cartilage [9, 13]. It is essential to compare the efficacy of multiple UTE techniques for the assessment of early degeneration in human cartilage in vivo, as well as investigate their underlying biochemical basis. The aim of this study is to elucidate the value of three commonly used UTE-MRI quantitative techniques in evaluating early cartilage degeneration, compare their diagnostic efficacy, and explore the possible pathological and biochemical basis of imaging results, guiding widespread clinical application and providing a theoretical basis.

## Materials and methods

### Participants

Twenty OA patients who underwent total knee arthroplasty (TKA) from June 2020 to August 2021 at Shanghai Tenth People’s Hospital of Tongji University were recruited. Exclusion criteria are as follows: general MRI contraindications (e.g., cardiac pacemakers, metal devices in the body), severe claustrophobia, orthopedic implants in the knee region, and secondary OA due to diseases such as tumors, immune diseases, or fractures.

This prospective study was approved by the Ethics Committee at Shanghai Tenth People’s Hospital of Tongji University (approval number: shsy-iec-ky-3964). Written informed consent was provided by all participants before MR examination.

### MRI protocol

MR imaging was performed one to three days before surgery using a transmit/receive 8-channel knee coil (Chenguang Medical Technologies, Shanghai, China) on a clinical 3.0 T MR scanner (Premier, GE Healthcare, Waukesha, WI, USA). To ensure optimal homogeneity of the magnetic field, the knee joint of OA patients and the coil were placed right in the center of the MRI scanner during the scan. Additionally, the scanned knee was positioned at the center of the coil. The following 5 imaging protocols were continuously performed without any clinical interventions: (A) three-dimensional (3D) UTE-MT imaging to measure UTE-MTR: off-resonance frequency = 2 kHz, saturation power = 750 degrees; (B) 3D UTE with AdiabT1ρ preparation to measure UTE-AdiabT1ρ value: spin-locking times = 2, 10, 30, and 60 ms; (C) fat-suppressed 3D multi-echo UTE imaging to measure single-component UTE-T2* value: TEs = 0.028, 4.9, 9.8, and 14.7 ms; (D) a 3D Cube Quant-T2 sequence to measure T2 value: TEs = 2, 4, 8, and 12 ms; (E) a 3D Cube Quant-T1ρ to measure T1ρ value: spin-locking times = 1, 10, 30, and 50 ms. Other imaging parameters included: field of view = 16 cm^2^, acquisition matrix = 256 × 256 pixels, slice thickness = 3 mm. The total scan time was approximately 41 min.

### Specimen preparation

After TKA, lateral femoral condyle and tibial plateau specimens were collected, which were less degenerated than weight-bearing areas, to ensure accurate measurements of biochemical content later. A total of 10 tibial plateaus and 15 lateral femoral condyles were obtained from the 20 participants, which were then embedded with saline-soaked gauze and placed in a −80 °C freezer for standardized processing.

**Fig. 1:**
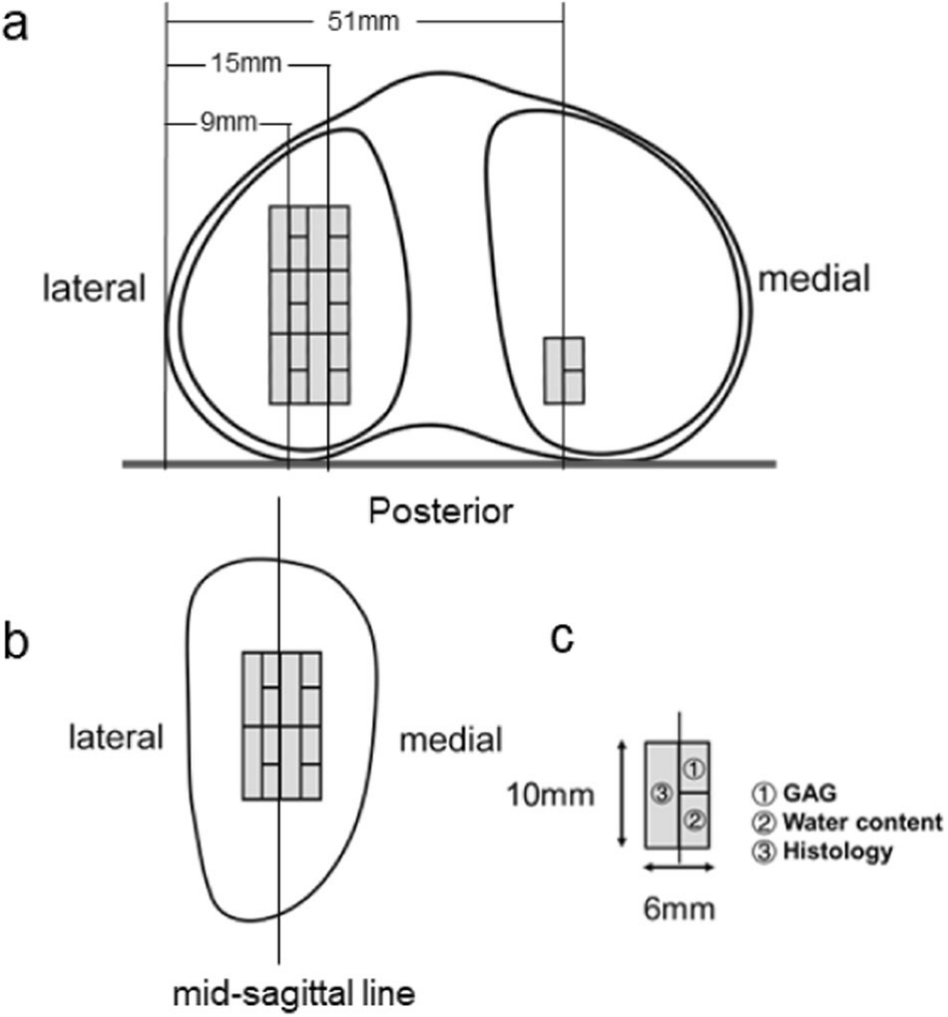
A schematic diagram of the sampling sites of the tibial plateau (**a**) and lateral posterior tibial condyle; each sample section (**c**). **a** The sampling sites, selected in accordance with the sagittal MRI slices, were set at 6-mm intervals orthogonal to the line connecting the edges of the medial and lateral posterior tibial condyles. Osteochondral samples were harvested from three sites on each slice (9 mm and 16 mm from the lateral edge of the lateral tibia condole) of the lateral tibia plateau and from one site on the slice (51 mm from the lateral edge of the lateral tibia condole) of the medial tibia plateau. **b** On the lateral femoral condyle, the four sampling sites centered on the intersection of the median sagittal line with the center line of the anterior-posterior border. **c** The sample size was 6 mm in width 10 mm in length. These samples were divided into three sections for GAG content, histological evaluation and water content

As with the sagittal MRI slices, the slices based on osteochondral sampling sites were set orthogonally to a line connecting the edge of the medial and lateral posterior tibial condyles (Fig. 1a). Specifically, the slices were located at distances of 9 mm, 15 mm, and 51 mm from the lateral edge of the lateral tibial condyle [14]. To determine the center of the middle two samples on the lateral platform, the midpoint of the anterior-posterior edge of the cartilage on the corresponding MRI sagittal image was used as a reference point. Four additional samples were placed adjacent to each other, anterior, and posterior to the middle two. In total, seven samples were collected from each tibial plateau, comprising six samples from the medial plateau and one sample from the posterior aspect of the lateral plateau. On the lateral femoral condyle, the midpoint of the anterior-posterior border of the cartilage was taken as a reference point, and four samples were collected around it (Fig. 1b). Each sample measured 6 mm in width × 10 mm in length and was divided into sections for GAG content, histological grades, and water content assessment, respectively (Fig. 1c). As a result of severe cartilage loss in some partial samples, 39 samples were excluded, and a total of 91 specimens were finally obtained.

### Quantitative measurement of GAG and water content

The cartilage in the corresponding area was excised with forceps and a razor blade. The cartilage sample solution for GAG content was prepared by digesting the cartilage with proteinase K. GAG content was quantified using the 1–9 dimethylmethylene blue (DMMB, Sigma–Aldrich) assay, as previously described [15, 16]. The wet weight was measured after removing the surface water from the cartilage, and the dry weight was recorded following roasting at 60 °C for three days. The water content was determined using the wet-to-dry ratio (W/D ratio).

### Histological analysis

Ninety-one cartilage samples were prepared by embedding and slicing them into 5-µm-thick sections, then staining with Safranin O-Fast Green (Sigma–Aldrich, St Louis, MO, USA) for histopathological or Sirius Red (Sigma–Aldrich, St Louis, MO, USA) for PLM collagen organization score (PLM-CO) analysis, respectively. A pathologist evaluated the samples according to Mankin score, which classified them into four groups based on the severity of cartilage degeneration: normal cartilage (grade 1 for scores 0–1, *n* = 17); mild degeneration (grade 2 for scores 2–5, *n* = 38); moderate degeneration (grade 3 for scores 6–9, *n* = 28); and severe degeneration (grade 4 for scores 10–14, *n* = 8). The integrity of collagen structure was assessed using the PLM-CO scores, which range from total disorganization (score 0) to healthy zonal architecture (score 5) [17].

### Image analysis

Digital Imaging and Communications in Medicine (DICOM) images obtained from the MR protocol mentioned above were analyzed in MatLab (Mathworks Inc., Natick, MA, USA). Regions of interests (ROIs) were manually drawn on the mid-sagittal section of the image of UTE-AdiabT1ρ and subsequently transferred to the remaining UTE-MRI sequence images. T2 and T1ρ values were measured using the T2 mapping software provided with the scanner.

Consistent with the location of obtaining cartilage specimens, ROIs were manually drawn in three consecutive layers before and after each sample measurement, and each ROI was measured 3 times by the same radiologist. The average value was taken for statistical analysis (Fig. S1).

### Statistical analysis

The software SPSS 20.0 (IBM Corp, Armonk, NY, USA) was conducted for the statistical analysis. As the data did not conform to normal distribution based on the Kolmogorov-Smirnov normality test, the variables were expressed using median and interquartile range (IQR). The Kruskal-Wallis test was used to compare the difference between quantitative UTE-MRI values and biochemical components among different Mankin grade groups. Intrareader reliability was described using the intra-class correlation coefficient (ICC). Spearman’s correlation coefficient was performed to determine the correlations between quantitative UTE-MRI values and Mankin scores, PLM-CO scores. Receiver operating characteristic (ROC) curves were used to evaluate the diagnostic efficacy of various quantitative UTE-MRI sequences for identifying mild cartilage degeneration. DeLong’s test was used for comparison of area under the curve (AUC). Retrospective power calculation was performed for ROC analysis and multivariable linear regression. In addition, multiple linear regression analysis was conducted to investigate the associations between the UTE-MRI techniques and biochemical components. Statistical significance was defined as a *p* value of less than 0.05.

## Results

### Volunteer demographic features

Twenty volunteers were recruited, including 7 males (age range: 68 to 78 years; 74 ± 3 years) and 13 females (age range: 57 to 79 years; 67 ± 6 years). Among them, 13 cases underwent MR examination of the left knee, and 7 cases underwent examination of the right knee. After TKA, 10 tibial plateaus and 15 lateral femoral condyles were collected; among them, 5 platforms and lateral condyles were from the same volunteer, other 5 platforms and 10 lateral condyles were from different volunteers. A total of 91 ROIs were included for statistical analysis.

### Comparison of quantitative UTE-MRI values and biochemical components between normal and mild cartilage degeneration

**Fig. 2:**
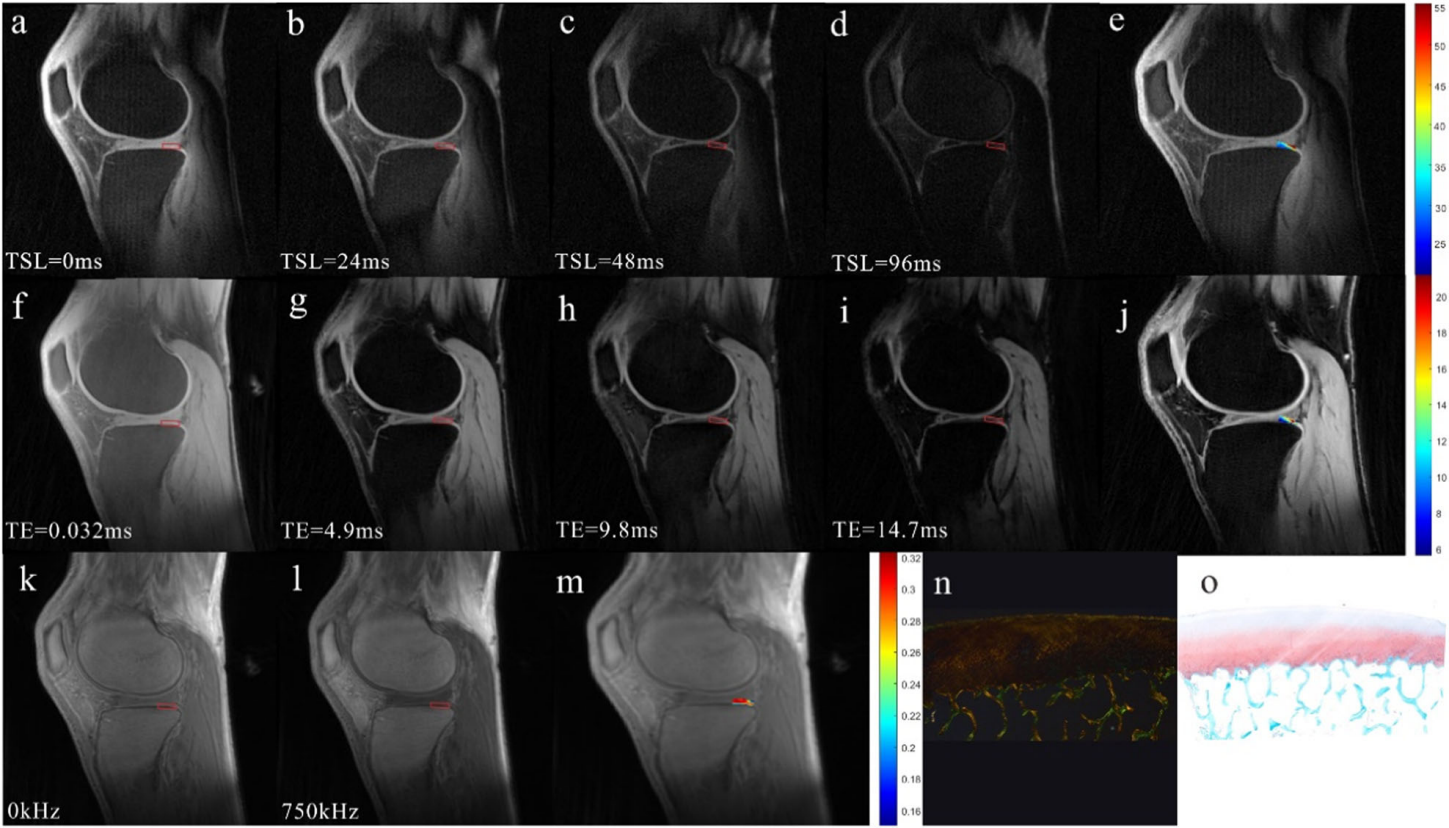
68 years old male with ROI classified as normal cartilage. **a**–**d** UTE-AdiabT1ρ images with different TSL times. **e** UTE-AdiabT1ρ map, corresponding to value of ROI = 35.66 ms. **f**–**i** UTE-T2* mapping images with different TE times. **j** UTE-T2* map, corresponding to value of ROI = 10.50 ms. **k**, **l** UTE-MT images with different saturation power. **m** UTE-MT map, corresponding to value of ROI = 29.03%. **n** PLM images corresponding to the ROI score 2. **o** Safranin O-Fast Green pathological stained section images corresponding to the ROI belong to the normal cartilage group (Mankin score = 1). UTE-MTR, ultrashort echo time-based magnetization transfer ratio; UTE-AdiabT1ρ, ultrashort echo time-based adiabatic T1ρ; UTE-T2*, ultrashort echo time-based T2*

**Fig. 3:**
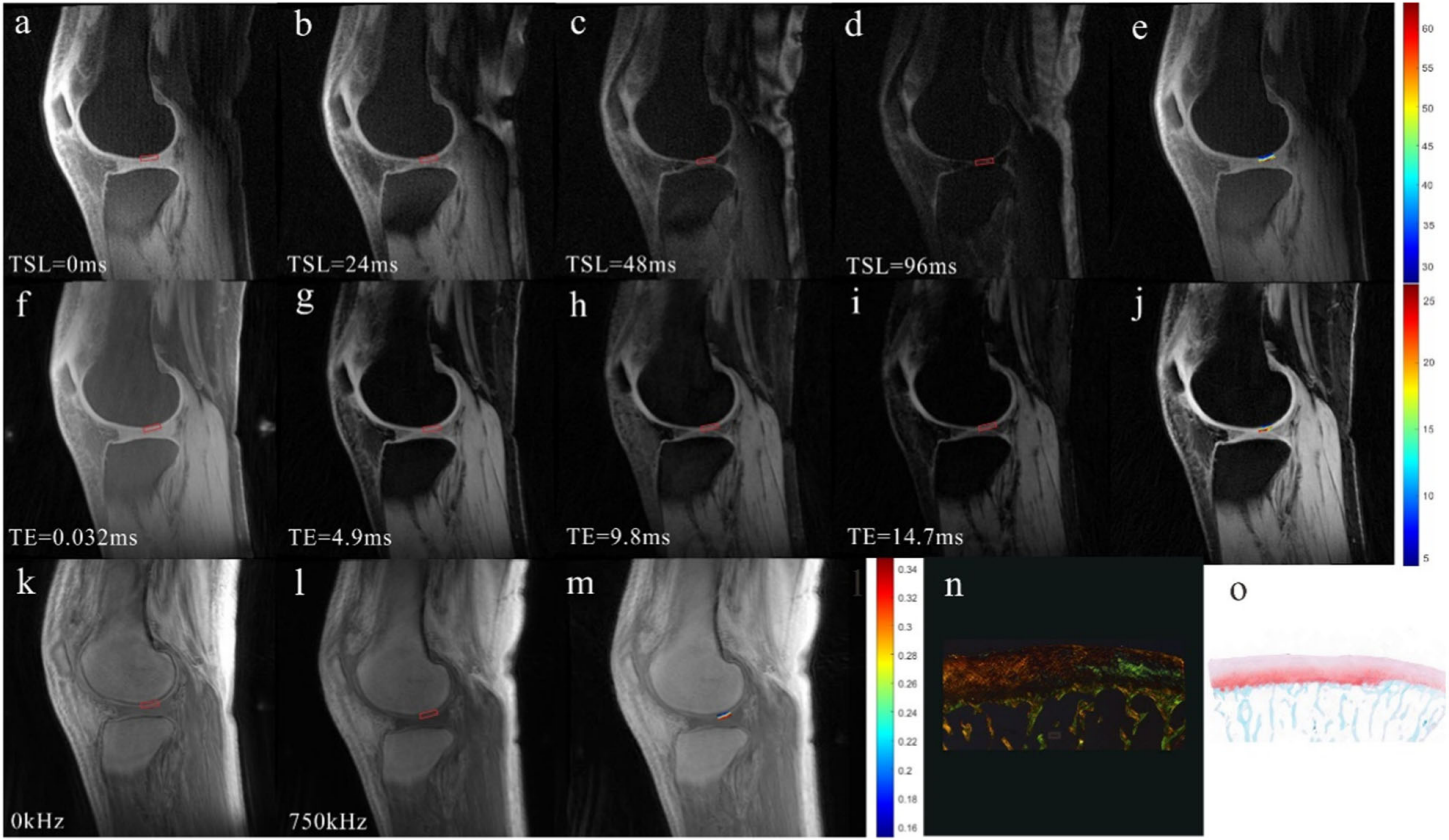
58-year-old female with ROI classified as mild degeneration of cartilage. **a**–**d** UTE-AdiabT1ρ images with different TSL times. **e** UTE-AdiabT1ρ map, corresponding to value of ROI = 47.66 ms. **f**–**i** UTE-T2* mapping images with different TE times. **j** UTE-T2* map, corresponding to of ROI = 14.96 ms. **k**, **l** UTE-MT images with different saturation power. **m** UTE-MT map, corresponding to value of ROI = 26.9%. **n** PLM images corresponding to the ROI score 4. **o** Safranin O-Fast Green pathological stained section images corresponding to the ROI belong to the mild degeneration cartilage group (Mankin score = 3). UTE-MTR, ultrashort echo time-based magnetization transfer ratio; UTE-AdiabT1ρ, ultrashort echo time-based adiabatic T1ρ; UTE-T2*, ultrashort echo time-based T2*

**Fig. 4:**
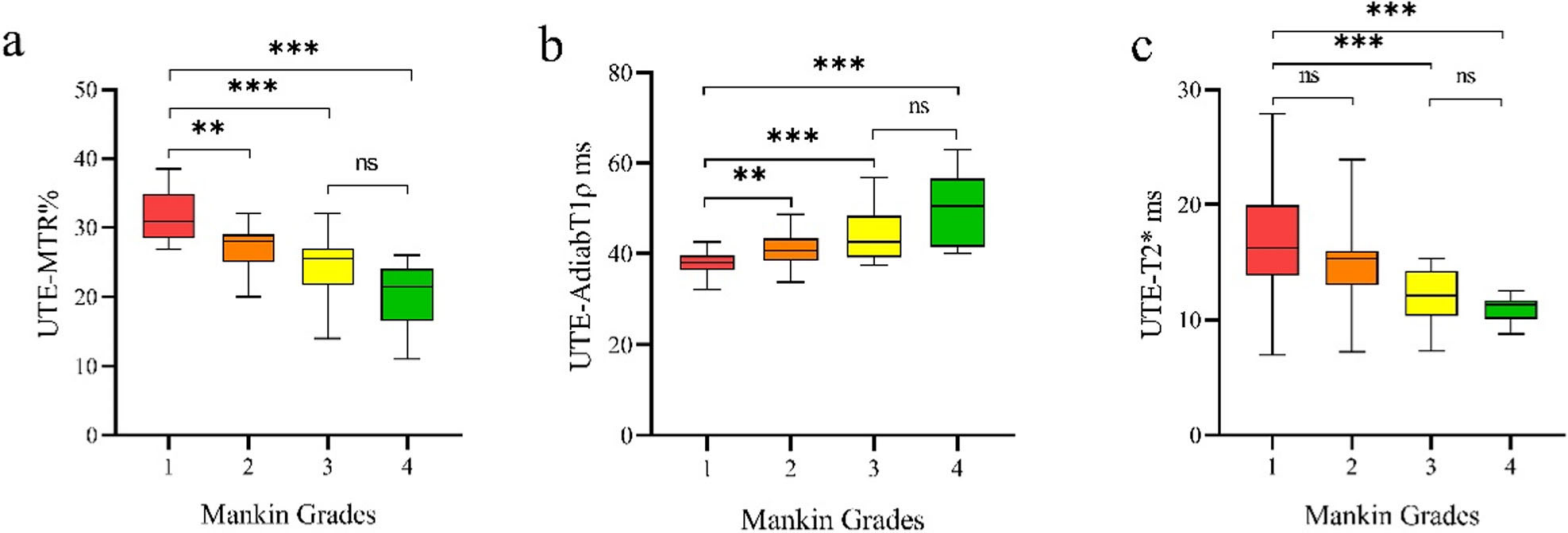
Box-plot diagrams illustrating quantitative UTE-MRI values among different Mankin grade groups. UTE-MTR, ultrashort echo time-based magnetization transfer ratio; UTE-AdiabT1ρ, ultrashort echo time-based adiabatic T1ρ; UTE-T2*, ultrashort echo time-based T2*. ns, nonsignificant; **0.001 ≤ *p* < 0.05; ***p < 0.001

Table 1 and Figs. 2–3 present the images and results of quantitative UTE-MRI values and biochemical components among different Mankin grade groups. As shown in Fig. 4, UTE-MTR and UTE-AdiabT1ρ values in the normal group differed from those in the mild degeneration group (*p* = 0.001, *p* = 0.002, respectively). The differences in UTE-T2* values, T2 values and T1rho values between normal and mild cartilage groups showed no significance (*p* = 0.19, *p* = 0.085, *p* = 0.124, respectively).

**Table 1:** The results of quantitative MRI examination among different Mankin grade groups

Quantitative MRI values/Biochemical components	Mankin Grade	*N*	Median (p25–p75)	*p* value		
				VS grade 2	VS grade 3	VS grade 4
UTE-AdiabT1ρ (ms)	1	17	37.85 (36.32–39.45)	0.002	< 0.001	< 0.001
	2	38	40.69 (38.50–43.36)		0.043	0.005
	3	28	43.05 (39.64–48.39)			0.141
	4	8	50.60 (41.55–56.59)			
UTE-MTR (%)	1	17	30.90 (28.45–34.95)	0.001	< 0.001	< 0.001
	2	38	28.00 (25.77–29.52)		0.012	0.001
	3	28	25.95 (22.12–27.07)			0.092
	4	8	21.80 (16.72–24.47)			
UTE-T2* (ms)	1	17	16.21 (13.83–19.94)	0.19	< 0.001	< 0.001
	2	38	15.30 (13.03–15.93)		< 0.001	0.001
	3	28	12.12 (10.34–14.21)			0.289
	4	8	11.33 (10.10–11.71)			
T1rho (ms)	1	17	41.17 (38.91–45.60)	0.124	0.002	0.001
	2	38	46.63 (40.09–51.29)		0.045	0.014
	3	28	51.02 (44.57–63.17)			0.258
	4	8	58.01 (54.16–64.71)			
T2 (ms)	1	17	35.44 (33.46–36.55)	0.085		
	2	38	37.33 (32.65–42.22)			
	3	28	39.49 (34.09–42.02)			
	4	8	45.44 (32.64–49.33)			
W/D ratio	1	17	4.08 (3.55–4.46)	0.442	< 0.001	< 0.001
	2	38	4.28 (4.09–4.46)		< 0.001	< 0.001
	3	28	4.89 (4.43–5.19)			0.335
	4	8	5.10 (4.66–5.40)			
GAG content (μg/mL)	1	17	287.50 (223.56–427.33)	0.001	< 0.001	< 0.001
	2	38	194.54 (167.79–228.23)		0.19	0.005
	3	28	155.35 (115.24–204.77)			0.194
	4	8	111.32 (93.49–171.39)			

*UTE-MRI* ultrashort echo time magnetic resonance imaging, *UTE-MTR* ultrashort echo time-based magnetization transfer ratio, *UTE-AdiabT1ρ* ultrashort echo time-based adiabatic T1ρ, UTE-T2* ultrashort echo time-based T2*

Intrareader reliability was excellent for all MRI value measurements. The UTE-MTR (ICC = 0.922, 95% confidence interval (CI): 0.891 to 0.945), UTE-AdiabT1ρ (ICC = 0.943, 95% CI: 0.920 to 0.960), UTE-T2* values (ICC = 0.930, 95% CI: 0.903 to 0.950), T1ρ values (ICC = 0.967, 95% CI: 0.954 to 0.977), and T2 values (ICC = 0.985, 95% CI: 0.978 to 0.989) all showed good reliability and reproducibility.

### Correlation analysis of quantitative UTE-MRI values and Mankin scores, as well as cartilage biochemical components and Mankin scores

**Fig. 5:**
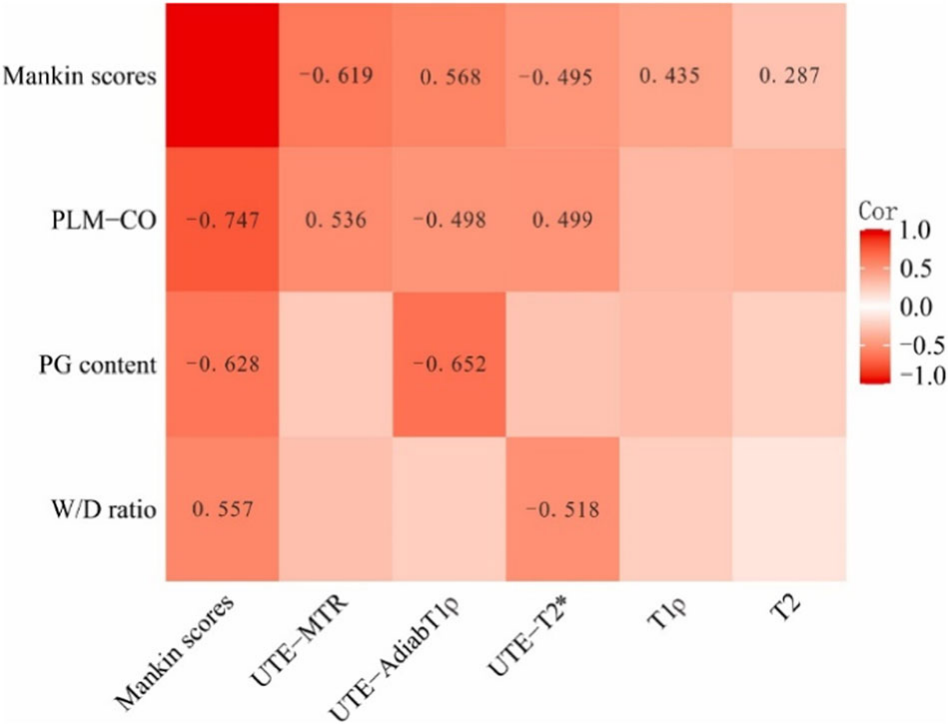
The correlation among quantitative MRI examination, Mankin scores and biochemical components. UTE-MTR values showed a moderate negatively correlation with Mankin scores (*r* = −0.619, *p* < 0.001). UTE-AdiabT1ρ values showed a moderate correlation with Mankin scores (*r* = 0.568, *p* < 0.001). The UTE-T2* values showed a low correlation with Mankin scores (*r* = −0.495, *p* < 0.001). T1ρ values showed low correlation with Mankin scores (*r* = 0.435, *p* < 0.001). The T2 values showed negligible correlation with Mankin scores (*r* = 0.287, *p* = 0.006). UTE-MTR values performed moderate correlation with PLM-CO (*r* = 0.536, *p* < 0.001), UTE-MTR, ultrashort echo time-based magnetization transfer ratio; UTE-AdiabT1ρ, ultrashort echo time-based adiabatic T1ρ; UTE-T2*, ultrashort echo time-based T2*

UTE-MTR values showed the highest correlation with Mankin scores (*r* = −0.619, *p* < 0.001) among quantitative MRI sequences (Fig. 5). UTE-T2* values and T1ρ values showed low correlation with Mankin scores (*r* = −0.495, *p* < 0.001, *r* = 0.435, *p* < 0.001, respectively). T2 values showed negligible correlation with Mankin scores (*r* = 0.287, *p* = 0.006). PLM-CO showed a high correlation with Mankin scores (*r* = −0.747, *p* < 0.001). GAG content and W/D ratio showed moderate correlation with Mankin scores (*r* = −0.628, *p* < 0.001, *r* = 0.557, *p* < 0.001).

### ROC curve analysis

**Fig. 6:**
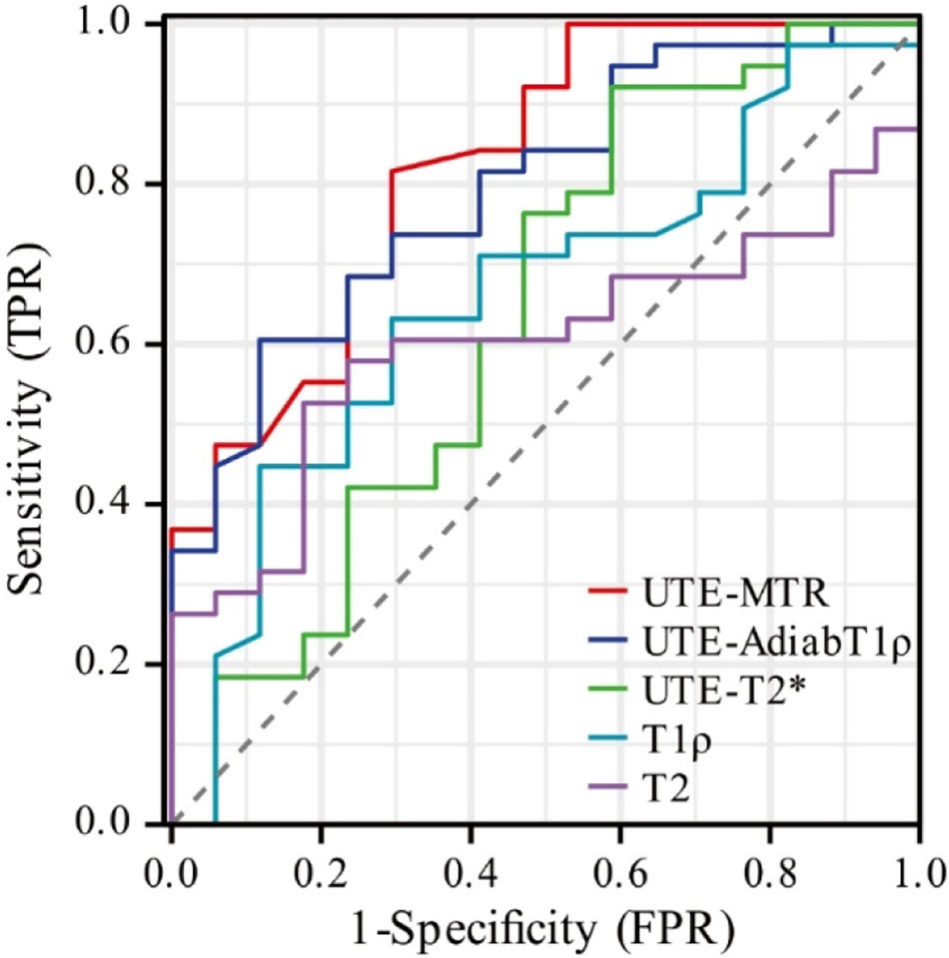
ROC curves of quantitative UTE-MRI sequences for the diagnosis of mild cartilage degeneration. The AUC of UTE-MTR (AUC = 0.824, 95% CI: (0.706–0.943); *p* < 0.001) and UTE-AdiabT1ρ (AUC = 0.796, 95% CI: (0.675–0.917), *p* = 0.001) were higher than that of UTE-T2* (AUC = 0.635, 95% CI: (0.461–0.808); *p* = 0.113). The AUC of T1ρ (AUC = 0.657, 95% CI: (0.501–0.813); *p* = 0.065) and T2 (AUC = 0.602, 95% CI: (0.454–0.750); *p* = 0.229) were both statistically insignificant. AUC, area under the curve; CI, confidence interval; ROC, receiver-operating characteristic; UTE-MRI, ultrashort echo time magnetic resonance imaging; UTE-MTR, ultrashort echo time-based magnetization transfer ratio; UTE-AdiabT1ρ, ultrashort echo time-based adiabatic T1ρ; UTE-T2*, ultrashort echo time-based T2*

As shown in Table 2 and Fig. 6, UTE-MT has an AUC of 0.824 (*p* < 0.001, 95% CI: (0.706–0.943)) and diagnostic sensitivity (0.816) for the diagnosis of mild cartilage degeneration. UTE-AdiabT1ρ had the highest diagnostic specificity (0.882) and positive predictive value (0.920) with an AUC of 0.796 (*p* = 0.001, 95% CI: (0.675–0.917)). Although UTE-T2* had the highest diagnostic sensitivity (0.921) and F1 score (0.843), its AUC was 0.635 (*p* = 0.113, 95% CI: (0.461–0.808)). DeLong’s test revealed that only AUC of UTE-MTR versus T2 values and UTE-AdiabT1ρ versus T2 values were significantly different (*p* = 0.0175, *p* = 0.0195, respectively). All the power values were < 0.05.

**Table 2:** Comparison of the diagnostic efficacy of quantitative MRI values for mild cartilage degeneration

MRI values	AUC	95% CI	*p*	sensitivity	specificity	PPV	NPV	F1 score
UTE-MTR	0.824	0.706–0.943	< 0.001	0.816	0.706	0.861	0.632	0.838
UTE-AdiabT1ρ	0.796	0.675–0.917	0.001	0.605	0.882	0.920	0.500	0.730
UTE-T2^*^	0.635	0.461–0.808	0.113	0.921	0.412	0.778	0.700	0.843
T1ρ	0.657	0.501–0.813	0.065	0.632	0.706	0.826	0.462	0.716
T2	0.602	0.454–0.750	0.229	0.526	0.823	0.870	0.438	0.656

*AUC* area under the curve, *CI* confidence interval, *ROC* receiver-operating characteristic, *PPV* positive predictive value, *NPV* negative predictive value, *UTE-MTR* ultrashort echo time-based magnetization transfer ratio, *UTE-AdiabT1ρ* ultrashort echo time-based adiabatic T1ρ, UTE-T2* ultrashort echo time-based T2*

### Multiple linear regression analysis of quantitative UTE-MRI sequences and biochemical components

UTE-MTR values performed moderate correlation with PLM-CO (*r* = 0.536, *p* < 0.001), low correlation with W/D ratio (*r* = −0.302, *p* = 0.004) and negligible correlation with GAG content (*r* = 0.251, *p* = 0.016). UTE-AdiabT1ρ values performed moderate correlation with GAG content (*r* = −0.652, *p* < 0.001), low correlation with PLM-CO (*r* = −0.498, *p* < 0.001) and negligible correlation with W/D ratio (*r* = 0.231, *p* = 0.028). UTE-T2* values performed moderate correlation with W/D ratio (*r* = −0.518, *p* < 0.001), low correlation with PLM-CO (*r* = 0.499, *p* < 0.001) and negligible correlation with GAG content (*r* = 0.281, *p* = 0.007). Multiple linear regression analysis showed that UTE-MTR values were significantly correlated with PLM-CO (*β* = 0.534, *p* < 0.001), but no correlation with GAG content (*β* = 0.095, *p* = 0.336) and W/D ratio (*β* = −0.019, *p* = 0.854), as shown in Table 3. UTE-AdiabT1ρ values were significantly correlated with GAG content (*β* = −0.236, *p* = 0.011) and PLM-CO (*β* = −0.500, *p* < 0.001), but no correlation with W/D ratio (*β* = 0.039, *p* = 0.679). UTE-T2* values were significantly correlated with W/D ratio (*β* = −0.304, *p* = 0.003), GAG content (*β* = 0.218, *p* = 0.027) and PLM-CO (*β* = 0.225, *p* = 0.043). All the power values were > 0.90.

**Table 3:** Correlation analysis between UTE-MRI sequences and biochemical components

UTE-MRI sequences	Correlation analysis		PLM-CO	GAG content	W/D ratio
UTE-MTR	Univariate analysis	Correlation coefficient	0.536	0.251	−0.302
		*p* value	< 0.001	0.016	0.004
	Multivariate analysis	Standardized partial regression coefficient (β)	0.534	0.095	−0.019
		*p* value	< 0.001	0.336	0.854
UTE-AdiabT1ρ	Univariate analysis	Correlation coefficient	−0.498	−0.652	0.231
		*p* value	< 0.001	< 0.001	0.028
	Multivariate analysis	Standardized partial regression coefficient (β)	−0.5	−0.236	0.039
		*p* value	< 0.001	0.011	0.679
UTE-T2*	Univariate analysis	Correlation coefficient	0.499	0.281	−0.518
		*p* value	< 0.001	0.007	< 0.001
	Multivariate analysis	Standardized partial regression coefficient (β)	0.225	0.218	−0.304
		*p* value	0.043	0.027	0.003

*UTE-MTR* ultrashort echo time-based magnetization transfer ratio, *UTE-AdiabT1ρ* ultrashort echo time-based adiabatic T1ρ, UTE-T2* ultrashort echo time-based T2*, *PLM-CO* polarized optical microscope collagen organization score, *W/D* ratio wet-to-dry ratio

## Discussion

Biochemical alterations of early cartilage degeneration existed in the extracellular matrix (ECM), including the loss of normal collagen network structure and reduction of PG, instead of cartilage fibrillation and fragmentation which happened in the advanced OA [18]. Given that these degenerative changes persist throughout the various stages of OA, collagen network structure and PG content are more appropriate as specific intrinsic biomarkers for detecting early cartilage degeneration, as opposed to water content, which is influenced by them [19]. It is found that three quantitative UTE-MRI sequences all showed a higher correlation with Mankin scores than T2 and T1ρ values. The advantage of ultra-short TE based on UTE-MRI scanning enables the full-layer evaluation of cartilage that cannot be displayed by conventional MRI scans [20]. According to our results, UTE-MTR, and UTE-AdiabT1ρ values were able to distinguish normal cartilage from mildly degenerated cartilage, while UTE-T2* and T2 and T1ρ values could not. Although T2 and T1ρ values can be used to evaluate degenerated cartilage, they are less sensitive to short T2 signals in the deeper layers of the cartilage [14, 21, 22]. In addition, the magic angle effect still had an impact on UTE-T2* and UTE-T1ρ quantitative analysis [23, 24]. The value influenced by the magic angle may obscure the effect of cartilage degeneration, thereby impacting the accuracy of the results. However, it has been confirmed that UTE-MT and UTE-AdiabT1ρ exhibited reduced sensitivity to the magic angle effect and will help to improve the robustness of quantitative UTE-MRI sequences [25, 26]. Thus, our results endorse the clinical value of the UTE-MRI sequence for early diagnosis of cartilage degeneration, specifically UTE-MT and UTE-AdiabT1ρ.

Almost all quantitative MRI examinations, except for T2 values, were capable of distinguishing between normal and severe, normal and moderate, mild and moderate, and mild and severe cartilage degeneration. As cartilage degradation gradually worsens, the changes in biochemical components within the cartilage become more significant, potentially leading to easier detection. And they all failed to identify moderate and severe cartilage degeneration, possibly due to severe damage to the superficial cartilage layer at advanced stages, resulting in MRI measurement bias. Furthermore, the limited participation of individuals with severe cartilage degeneration may impact the outcomes.

What’s more, the correlation analysis between quantitative UTE-MRI values and biochemical components revealed that the measured UTE-MTR in vivo reflected collagen network structure while the measured UTE-AdiabT1ρ values in vivo were more sensitive to changes in PG content. Li et al [27] reported a significant correlation between MTR and collagen degradation as well as loss of PG. In our previous study, Shao et al [28] demonstrated moderate correlation between UTE-MTR values and PLM-CO scores in vitro, which is consistent with our in vivo study. Wu et al [25] evaluated the feasibility of 3D UTE-Cones-AdiabT1ρ for in vivo assessment of whole knee cartilage in patients. Wan et al [29] claimed that UTE-AdiabT1ρ values are capable of detecting enzymatic PG loss in extracorporeal human cartilage specimen and may have more sensitivity to PG loss than other UTE biomarkers. In our study, UTE-AdiabT1ρ values demonstrated moderate correlation both with GAG content and PLM-CO. Hafner et al [30] reported that aberrant loading-induced changes in T1ρ reflected severe matrix changes in human cartilage. Variations in T1ρ values can result from several factors in addition to proteoglycan depletion, such as collagen fiber orientation and the water content attached to them. The meshwork of collagen fibrils combined with the proteoglycan gel to traps water. The disruption of collagen network and the loss of PG are not separate processes but mutually reinforcing results [31]. In the multiple linear regression analysis of UTE-AdiabT1ρ values, the PLM-CO scores even accounted for a higher percentage than the GAG content. It was found that three biochemical components all affected UTE-T2* values, and water content had the greatest impact. This may be why UTE-T2* values failed to distinguish normal and mild cartilage degeneration, but performed well in mild to moderate and mild to severe cartilage degeneration. It’s worth noting that PLM-CO scores affected all three quantitative UTE-MRI values through multiple linear regression. This finding underscores the significance of detecting collagen integrity for early cartilage degeneration, and UTE-MTR with the highest correlation to collagen integrity has a definite advantage in diagnosing early cartilage degeneration with great clinical potential. Both our study and Shao’s discovered that UTE-MTR had a highest diagnostic efficacy for mild cartilage degeneration through ROC curves and exhibited the highest correlation with Mankin scores, highlighting its potential for early cartilage degeneration detection in clinical applications.

There are some limitations in our study. First, although the same radiologist strived to achieve point-to-point correspondence between knee joint cartilage ROI outlining and obtaining cartilage specimens, it is inevitable that there may have been errors. The statistical results of 91 ROIs minimized these errors. Second, although the total number of cartilage samples was 91, the number of participants is relatively small, and the representativeness of the results is limited. For ROC analysis, all the power values were < 0.05, which we believe may be related to the fact that ROC analysis was only compared in two groups. However, the power of the multivariable linear regression showed that our sample size has good diagnostic efficacy (all power > 0.90). In the future, more volunteers will be recruited and mechanical properties research related to cartilage will be added.

In conclusion, quantitative UTE-MRI values have shown greater in vivo diagnostic value for early cartilage degeneration compared to conventional T2 and T1ρ values. Of them, UTE-MTR has the highest potential in diagnosing early cartilage degeneration. UTE-MTR, UTE-AdiabT1ρ, and UTE-T2* values respectively mainly reflect different aspects of cartilage degeneration, that is, the integrity of collagen structure, PG content, and water content, although there are cross effects between them. Quantitative UTE-MRI parameters have the potential to serve as imaging biomarkers of early cartilage degeneration by reflecting the changes in biochemical components, thereby aiding in the early detection of early cartilage degeneration in clinical settings.

### Supplementary information

ELECTRONIC SUPPLEMENTARY MATERIAL

## Data Availability

The datasets used and/or analyzed during the current study are available from the corresponding author on reasonable request.

## Abbreviations

AUC: Area under the curve
GAG: Glycosaminoglycan
MRI: Magnetic resonance imaging
MTR: Magnetization transfer ratio
OA: Osteoarthritis
PG: Proteoglycan
ROC: Receiver operating characteristic
ROI: Region of Interest
TE: Time of echo, TE
TKA: Total knee arthroplasty
UTE: Ultrashort time of echo
