# Supplementary material for: A feasibility study of in vivo quantitative ultra-short echo time-MRI for detecting early cartilage degeneration
Source: Insights Imaging. 2024 Jun 26;15:162. doi: 10.1186/s13244-024-01734-4 (PMC11208376; doi:10.1186/s13244-024-01734-4)
Supplement: Supplementary file 1 — ELECTRONIC SUPPLEMENTARY MATERIAL [file 13244_2024_1734_MOESM1_ESM.pdf]

A feasibility study of in vivo quantitative ultra-short echo time-MRI for detecting early cartilage degeneration

ELECTRONIC SUPPLEMENTARY MATERIAL

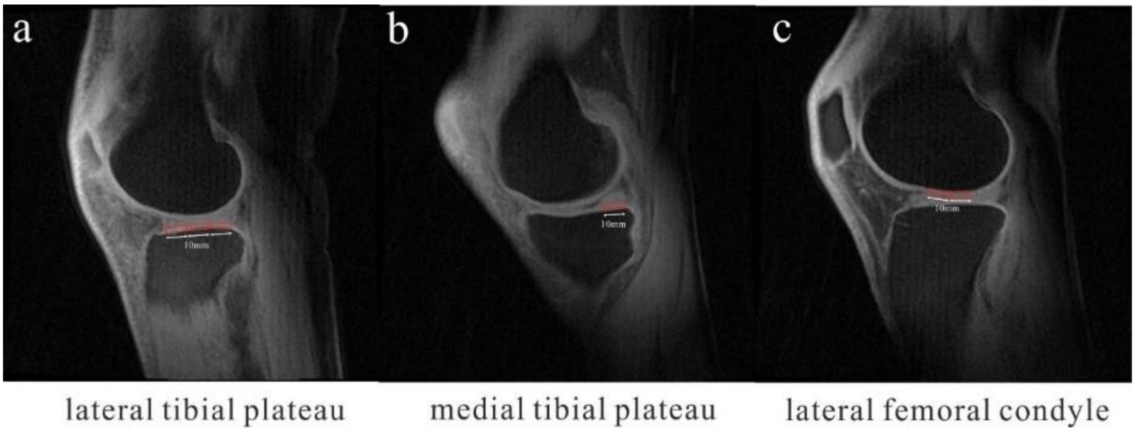

Figure S1. Schematic drawing of the ROIs manually on UTE-AdiabT1rho (TSL=0ms): (a) ROIs on the lateral tibial plateau corresponding to the sample. (b) ROIs on the medial tibial plateau posterior to the sample. (c) ROIs on the lateral femoral condyle corresponding to the sample.UTE-AdiabT1ρ, ultrashort echo time-based adiabatic T1ρ; ROI: Regions of interests.
